# Preparation of hospitals for mass casualty incidents in Bavaria, Germany: care capacities for penetrating injuries and explosions in TerrorMASCALs

**DOI:** 10.1186/s13049-021-00970-7

**Published:** 2021-10-30

**Authors:** Nina Thies, Alexandra Zech, Thorsten Kohlmann, Peter Biberthaler, Michael Bayeff-Filloff, Karl-Georg Kanz, Stephan Prückner

**Affiliations:** 1 Institut für Notfallmedizin und Medizinmanagement (INM), Klinikum der Universität München, LMU München, Schillerstr. 53, 80336 Munich, Germany; 2 Department of Trauma Surgery, University Hospital rechts der Isar, Technical University Munich, Munich, Germany; 3 Bayerisches Staatsministerium des Innern für Sport und Integration, Ärztlicher Landesbeauftragter Rettungsdienst, Munich, Germany

**Keywords:** Terrorist attack, Trepanation, Thoracotomy, Trauma center

## Abstract

**Background:**

In a terror attack mass casualty incident (TerrorMASCAL), compared to a “normal” MASCAL, there is a dynamic course that can extend over several hours. The injury patterns are penetrating and perforating injuries. This article addresses the provision of material and personnel for the care of special injuries of severely injured persons that may occur in the context of a TerrorMASCAL.

**Methods:**

To answer the research question about the preparation of hospitals for the care of severely injured persons in a TerrorMASCAL, a survey of trauma surgery departments in Bavaria (Germany) was conducted using a questionnaire, which was prepared in three defined steps based on an expert consensus. The survey is divided into a general, neurosurgical, thoracic, vascular and trauma surgery section. In the specialized sections, the questions relate to the implementation of and material and personnel requirements for special interventions that are required, particularly for injury patterns following gunshot and explosion injuries, such as trepanation, thoracotomy and balloon occlusion of the aorta.

**Results:**

In the general section, it was noted that only a few clinics have an automated system to notify off-duty staff. When evaluating the data from the neurosurgical section, the following could be established with regard to the performance of trepanation: the regional trauma centers do not perform trepanation but nevertheless have the required material and personnel available. A similar result was recorded for local trauma centers. In the thoracic surgery section, it could be determined that almost all trauma centers that do not perform thoracotomy have the required material available. This group of trauma centers also stated that they have staff who can perform thoracotomy independently. The retrograde endovascular aortic occlusion procedure is possible in 88% of supraregional, 64% of regional and 10% of local trauma centers. Pelvic clamps and external fixators are available at all trauma centers.

**Conclusion:**

The results of the survey show potential for optimization both in the area of framework conditions and in the care of patients. Consistent and specific training measures, for example, could improve the nationwide performance of these special interventions. Likewise, it must be discussed whether the abovementioned special procedures should be reserved for higher-level trauma centers.

## Background

During a terrorist attack mass casualty incident (TerrorMASCAL), compared to a “normal” MASCAL, there is a dynamic course that can extend over several hours. In addition, there is the possibility that casualties may occur at different locations, and critical infrastructures may also be affected [1]. Injury patterns primarily involve penetrating and perforating injuries, which can result in rapid blood loss. As a result, the number of severely injured people is increased in a TerrorMASCAL [2].

An example of typical injury patterns after explosions can be obtained from data from the Gregorio Maranon University General Hospital (GMUGH), a hospital with 1800 beds, which was at the center of the 2004 Madrid bomb explosions in terms of victim care. Within the first 24 h, 37 interventions were performed in the operating theatre on 34 patients. The three most common surgical interventions were orthopedic (40%), visceral (18.9%) and neurosurgical (16.2%) in nature [3].

An analysis of emergency data from the Chris Hani Baragwanath Academic Hospital (CHB) in Johannesburg, South Africa, was used to provide an example of typical injuries after stabbing and gunshot wounds. This analysis showed that, among stabbing injuries, thoracic injuries were the most common (44.4%), whereas among gunshot injuries, abdominal injuries dominated (34.4%) and thoracic injuries came second (24.8%) [4].

The challenge is to transport patients with penetrating injuries to appropriate hospitals that can adequately care for these injury patterns [4, 5].

The following indications for the performance of special procedures corresponding to the injury patterns of a TerrorMASCAL are described in the German medical guidelines.

In both the S3 Polytrauma guideline and the Craniocerebral Trauma in Adulthood guideline, a life-threatening intracranial injury is an indication for emergency surgical intervention (recommendation grade A) [5, 6]. The S3 Polytrauma guideline calls for immediate thoracotomy in cases of penetrating thoracic injuries, severe hemodynamic shock, signs of pericardial tamponade and severe hemorrhage, in the absence of peripheral pulses, and in cases of cardiac arrest, provided that initial vital signs were observed or the onset of cardiac arrest was no longer than 5 min prior [5]. Emergency thoracotomy is also an integral part of the peri-arrest algorithm for trauma patients in the European Research Council (ERC) guideline [7]. In the S3 Polytrauma guideline, the treatment of fractures of the lower and upper extremities by means of an external fixation system is recommended if the condition does not allow definitive fracture stabilization; this applies in particular to patients with circulatory instability [5]. Later, definitive fracture treatment in unstable patients with limb injuries was also advocated in work by Taeger et al. A suitable initial treatment should be carried out by means of an external fixator [8, 9]. The identification of life-threatening limb bleeding and implementation of appropriate bleeding control measures, such as the use of tourniquets, is also recommended according to Advanced Trauma Live Support (ATLS®) [10].

Education in trauma care in Germany is organized by the trauma network of the German Society of Trauma Surgery (Deutsche Gesellschaft für Unfallchirurgie; DGU). The trauma network project was initiated in 2006, thereby enabling and improving qualified care for severely injured people throughout Germany and creating a basis for further development. All hospitals providing care for severely injured patients are certified and divided into three tiers of care (local, regional and supraregional). Depending on the severity of the patient’s injury, he or she can thus be sent to a suitable trauma center. This three-tier system is similar to the trauma system of the United States (US), with local trauma centers equal to US level III centers, regional trauma centers equal to US level II centers and supraregional trauma centers equal to US level I centers [11, 12].

The federal state of Bavaria currently has approximately 13.08 million residents in an area of 70,550 square kilometers [13]. According to the Bavarian Hospital Plan, Bavaria has more than 400 hospitals and six university hospitals. This means that the state of Bavaria has > 73,000 full inpatient beds and > 4000 partial inpatient beds [14]. At the time of the survey, 19 supraregional, 32 regional and 52 local trauma centers were registered in Bavaria.

By means of a survey, trauma surgery departments in the federal state of Bavaria (Germany) were questioned in extensive research to determine which material and personnel resources are available and could be used to treat specific injury patterns in severely injured persons after terrorist and amok attacks.

## Methods

To answer the research question about the preparation of hospitals for the care of severely injured persons in a TerrorMASCAL, a survey was conducted by means of a questionnaire.

This questionnaire was prepared in three defined steps based on an expert consensus. In the first round, the questionnaire was drafted by experts in the fields of anesthesia and emergency medicine from the Institute of Emergency Medicine and Medical Management (INM). In the next step, this draft was expanded and revised by experts in the fields of surgery and emergency medicine with Bavaria-wide medical responsibility. Finally, the questionnaire was checked and adapted with regard to test-psychological quality criteria.

All hospitals providing basic, specialized and maximum care as well as university hospitals and specialist hospitals with surgical care capacity that are included in the Bavarian Hospital Plan for acute care of the population were taken into account in the survey [14]. This means that all trauma centers throughout Bavaria are included.

A total of 193 surveys were sent by post to the chief physicians of the Departments of Trauma Surgery via the Institute for Emergency Medicine and Management in Medicine, University Hospital LMU Munich (Institut für Notfallmedizin und Medizinmanagement; INM).

The survey was conducted in November/December 2017 on a voluntary basis. After 14 days, a reminder email was sent to the departments. The data were processed in anonymized form, which means that the data cannot be used to trace participating hospitals in retrospect.

The survey comprises 28 questions and is divided into a general section and a neurosurgical, thoracic, vascular and trauma surgery section (Table 1).

**Table 1:** Structure of the survey

*General section*
Trauma center category
Distance between helipad, CT facility and trauma room
Method for alerting off-duty staff
*Neurosurgery section*
"What is the process for the care of a patient with severe traumatic brain injury at your hospital?"
Whether trepanation is performed
Material and personnel supply; time interval until the person performing the intervention is available
Possibility of subsequent monitoring (ICP/EVD)
*Thoracic surgery section*
"What is the process for the care of a patient with a penetrating chest injury and circulatory arrest?"
Whether an exploratory thoracotomy is performed in the trauma room (using an anterolateral approach in the supine position or clamshell thoracotomy)
Material and personnel supply; time interval until the person performing the intervention is available
Presence of a cardiac surgery unit and a thoracic surgery unit
*Vascular surgery section*
Whether balloon occlusion of the aorta is performed using the REBOA technique in patients with massive intraabdominal or pelvic bleeding
Material supply for the REBOA technique
Presence of a vascular surgery unit
*Trauma surgery section*
Number of pelvic cradles or pelvic slings
Number of tourniquets
Number of external fixation systems

The first question serves to classify the hospital according to the trauma center category (local, regional, supraregional) to be able to compare answers between the individual trauma center categories in subsequent sections of the survey. In the general section, the spatial framework conditions are recorded, especially in the case of in-hospital emergencies and diagnostics. In addition, the survey asks how long it takes staff to arrive at the hospital when they are alerted during off-duty hours and whether there are differences in the method for alerting off-duty staff among different levels of care.

Subsequently, specific care options are queried in the subject-specific sections to determine which trauma center category can fully provide care for these injury patterns.

Regarding neurosurgical care, questions are used to determine how many hospitals are able to treat patients with severe craniocerebral trauma, by means of trepanation if necessary, and to provide further care.

The thoracic and vascular surgery sections address the treatment of patients with injuries to the trunk of the body using the so-called clamshell technique and the retrograde endovascular aortic occlusion (REBOA) technique, respectively.

The trauma surgery section is used to determine the number of tourniquets, pelvic clamps and external fixation systems that are kept in the hospitals to obtain an overview of how many patients with the corresponding injury pattern can be treated.

The analysis was carried out with Microsoft Excel version 15.27 (Microsoft Corporation, Redmond, Washington, USA). For questions with an open answer format or multiple answers, the more conservative indication was always taken into account.

The study was approved by the ethics committee of the medical faculty of the University of Munich (Project-No.: 17-232).

## Results

A total of 193 surveys were sent out, including 19 to supraregional, 32 to regional, and 52 to local trauma centers and 90 to hospitals that were not certified accordingly. In all, 99/193 (51%) surveys were returned, three of which were incomplete. These were not taken into account, so 96/193 (50%) surveys were considered in the evaluation. In the presentation of the results, the number of hospitals and the percentage in brackets (rounded) are indicated.

**Fig. 1:**
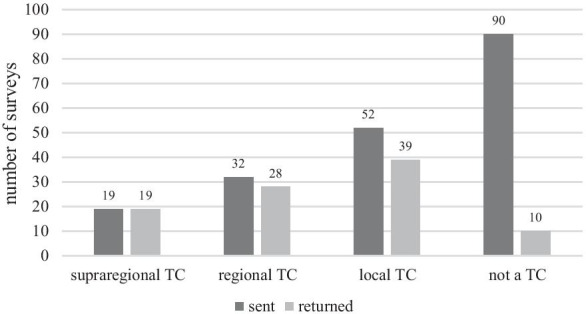
Survey response rates. *TC* trauma center

The response rate was broken down as follows: supraregional trauma centers, 19/19 (100%); regional trauma centers, 28/32 (88%); local trauma centers, 39/52 (75%); and remaining hospitals, 10/90 (11%) (Fig. 1). Since the response rate of the non-trauma-certified hospitals was so low, they were not included in the analysis.

**Fig. 2:**
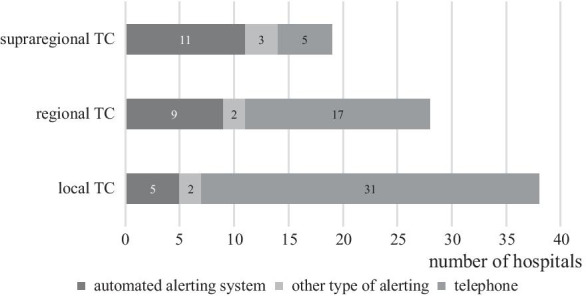
Systems for alerting off-duty personnel. Proportion of hospitals with/without an external alerting system. *TC* trauma center

Figure 2 shows how the hospitals notify off-duty staff (an external alerting system refers to an automated system for alerting medical staff). For the answer option *Other type of alerting system*, the possibility of a free text response was provided; the following free text responses were noted here: additional radio system, WhatsApp group, alerting with SMS, alarm server and internal telephone system.

The following results were noted in the neurosurgical section: trepanation is performed at all 19/19 (100%) supraregional trauma centers, as well as at 23/28 (82%) of regional trauma centers, but at only (17/39 or 44%) of local trauma centers.

Table 2 shows in more detail how trepanation is performed at regional and local trauma centers. Of the trauma centers that perform trepanation, 2/28 (7%) stated that they have no staff who can perform the procedure independently and on their own. However, according to the responses, almost all regional trauma centers that do not perform trepanation have the required material and personnel available.

**Table 2:** Performance of trepanation taking into account required material and personnel in regional and local trauma centers

	RTC		LTC	
	Yes	No	Yes	No
Performance of trepanation	23/28 (82%)	5/28 (18%)	17/39 (44%)	22/39 (56%)
Material stocking				
Yes	23/28 (82%)	4/28 (14%)	17/39 (44%)	9/39 (23%)
No		1/28 (4%)		13/39 (33%)
Personnel performing a trepanation independently				
Yes	21/28 (75%)	4/28 (14%)	17/39 (44%)	11/39 (28%)
No	2/28 (7%)	1/28 (4%)		11/39 (28%)
Specialization of individuals performing trepanation				
NS	10/28 (36%)	3/28 (11%)	3/39 (8%)	1/39 (3%)
NS + TS	8/28 (29%)	1/28 (4%)	7/39 (18%)	1/39 (3%)
NS + TS + other	1/28 (4%)			
TS	4/28 (14%)		7/39 (18%)	8/39 (21%)
TS + other				1/39 (3%)
Personnel workability				
Within*	23/28 (82%)	4/28 (14%)	16/38 (42%)	11/39 (28%)
Outside of*	23/28 (82%)	3/28 (11%)	16/38 (42%)	11/39 (28%)

*RTC* regional trauma center, *LTC* local trauma center, *NS* neurosurgery, *TS* trauma surgery

*Regular working hours

Again, 9/39 (23%) local trauma centers do not perform trepanation but have both the required material and personnel, while 2/39 (5%) reported having the required personnel only.

Further patient monitoring according to the intracranial pressure (ICP) and external ventricular drainage (EVD) is possible at all supraregional trauma centers 19/19 (100%), at 21/28 (75%) of regional trauma centers, but at only 5/39 (13%) of local trauma centers.

The thoracic surgery section contains questions about the performance of an exploratory thoracotomy on site in the trauma room (by means of anterolateral access in the supine position or clamshell thoracotomy). Table 3 shows that almost all trauma centers that do not perform thoracotomy keep the required material on hand and store it in the operating theatre (and not in the trauma room). This group of trauma centers also indicated that they have the required material and personnel who can perform a thoracotomy independently. Table 4 indicates the time until the person responsible for performing the procedure is available within and outside of regular working hours.

**Table 3:** Performance of a thoracotomy with provision of material and personnel as well as the location of material storage

	STC		RTC		LTC	
	Yes	No	Yes	No	Yes	No
Performance of thoracotomy	13/19 (68%)	6/19 (32%)	15/27 (56%)	12/27 (44%)	18/38 (47%)	20/38 (53%)
Material stocking						
Yes	13/19 (68%)	6/19 (32%)	15/27 (56%)	11/27 (41%)	17/38 (45%)	15/38 (39%)
No				1/27 (4%)	1/38 (3%)	5/38 (13%)
How the material is stored						
Individual instruments				1/27 (4%)	1/32 (3%)	3/32 (9%)
Complete set	13/19 (68%)	6/19 (32%)	14/26 (54%)	11/26 (42%)	16/32 (50%)	12/32 (38%)
Location of material storage						
Trauma room	7/18 (39%)	1/18 (6%)	9/27 (33%)	1/27 (4%)	6/31 (19%)	1/31 (3%)
Trauma room + operating theatre	3/18 (17%)		3/27 (11%)		2/31 (6%)	1/31 (3%)
Operating theatre	2/18 (11%)	5/18 (28%)	3/27 (11%)	11/27 (41%)	8/31 (26%)	13/31 (42%)
Personnel performing a thoracotomy independently						
Yes	13/19 (68%)	6/19 (32%)	15/27 (56%)	12/27 (44%)	18/35 (51%)	14/35 (40%)
No						3/35 (9%)

*STC* supraregional trauma center, *RTC* regional trauma center, *LTC* local trauma center

**Table 4:** Results

	STC	RTC	LTC
*Thoracic surgery section*			
How much time does it take for the person who performs the intervention to be ready within* (in min)			
Median	0	5	10
n	17	26	32
How much time does it take for the person who performs the intervention to be ready outside of* (in min)			
Median	15	20	30
n	16	27	31

*STC* supraregional trauma center, *RTC* regional trauma center, *LTC* local trauma center

*Regular working hours

The number of available tourniquets, external fixation systems and pelvic clamps/pelvic slings are shown in Table 5. The total was always taken into account without differentiation between large and small external fixator sets.

**Table 5:** Material storage for vascular surgery/trauma surgery

	STC	RTC	LTC
Pelvic sling/pelvic clamps			
Median	5	2.5	2
n	18	28	39
Tourniquets			
Median	10	5	2
n	16	25	32
External fixation systems			
Median	5	5	3
n	19	27	39

*STC* supraregional trauma center, *RTC* regional trauma center, *LTC* local trauma center

## Discussion

The objective of this survey was to obtain an overview of the care capacities in the event of a mass casualty incident following a terrorist and similar attack with specific penetrating injury patterns in the federal state of Bavaria (Germany).

The survey response rate shows a clear difference between trauma centers 86/103 (83%) and non-trauma-certified hospitals 10/90 (11%). Three non-trauma-certified hospitals provided feedback that they did not feel addressed due to the specific question and lack of material availability. This could also have been a reason for the lack of a response from other non-trauma-certified hospitals and thus may explain the significantly lower response rate of these institutions.

### Personnel availability

Unlike a standard MASCAL, the arrival of patients in a TerrorMASCAL is dynamic, which makes it difficult to plan the first minutes and hours; thus, in this situation, hospitals benefit from recruiting personnel as quickly as possible [1]. The number of self-referrals who visit a hospital in a MASCAL situation who have not received prior (pre-) triage or first aid should not be underestimated. Thus, the lead time for hospitals to notify additional, needed personnel in such a MASCAL, is limited [15]. For these reasons, an automated alerting system is very helpful in saving valuable time in the preparation phase. The survey revealed that only slightly more than half (11/19, 58%) of the supraregional trauma centers, approximately one-third (9/28, 32%) of the regional trauma centers, and 5/38 (13%) of the local trauma centers indicated that they used an automated alerting system.

In all, 5/19 (26%) of the supraregional, 17/28 (61%) of the regional and 31/38 (82%) of the local trauma centers stated that they used a telephone alerting system, although this requires a large amount of time and the hospital is only fully able to act during on-call hours as soon as sufficient staff are available. At this point, the question arises as to whether the hospitals use automatic systems, which is not addressed by the answer options.

### Performance of trepanation

Emergency trepanation is required if indicated by guidelines. All supraregional trauma centers, as well as 23/28 (82%) of the regional and almost half (17/39, 44%) of the local trauma centers, stated that they perform this procedure. However, the possibility for further monitoring by means of an ICP/EVD system is not standard at any of these regional or local centers.

The guidelines call for the primary care of severely injured patients in a suitable (regional or supraregional) trauma center because of the reduction in lethality; if this is not possible within 30 min, a closer facility is to be approached for initial care, and if necessary, a secondary transfer is to be carried out. However, this presupposes both admission capacity at other facilities and the availability of suitable transport capacity using adequate rescue equipment. However, both factors can be severely limited under the special conditions of a MASCAL. This differs from the highly centralized US system [5, 6, 12].

### Who can perform special interventions?

Trepanation is not performed at 4/28 (14%) of the regional and 9/39 (23%) of the local trauma centers, even though these centers store the required material and have personnel who can perform trepanation independently. It is striking that at 3/28 (11%) of the regional trauma centers performing trepanation, the performing specialist discipline is exclusively neurosurgery. Furthermore, these centers indicate that they cooperate with other hospitals that provide appropriately qualified physicians for neurosurgical measures. One comment indicates that only the chief physician can perform the procedure; another comment indicates that trepanation has not been performed in recent years. It can be concluded from these results that the feasibility at regional trauma centers, and especially at local trauma centers, is strongly dependent on the person performing the intervention.

One possible cause could be the change in the training regulations for surgeons [16].

In the past, surgeons in Germany were trained in all areas of surgery and acquired broad basic knowledge for surgery in general. This basic surgical training used to last six years, whereas now it only lasts two years. The training regulations thus provide for earlier specialization, e.g., as a visceral, trauma or thoracic surgeon. This limits the diversity of the skills of a surgeon [16, 17].

This explains why the material for special procedures is still present but the knowledge required for its-application is no longer standard.

Moreover, a lack of routine in performing these interventions might be more likely to contribute to a refusal to perform them in a situation that is not commonplace [18]. The picture is similar for thoracotomy. The requirements for a thoracotomy in the operating theatre (e.g., for elective surgery) are fulfilled, but the procedure is not carried out in the trauma room when there is an emergency indication.

To adequately prepare staff for these special interventions, especially in a MASCAL, they may benefit from participating in special short courses, such as the Advanced Surgical Skills for Exposure in Trauma (ASSET) course [19] or the Prehospital and Emergency Department Thoracotomy (PERT) course for performing a thoracotomy [20].

For the London and Australian Air Rescue teams, training in an algorithm for performing an emergency thoracotomy is an integral part of their training [21, 22].

Corresponding courses, as a fixed component of further training, should be offered and carried out throughout the country and could help to better integrate existing but unused material and personnel resources.

### Emergency thoracotomy in the trauma room/operating theatre

In the case of an emergency thoracotomy in the trauma room, there is a clear discrepancy between the requirements in the guidelines and feasibility at the hospitals. The procedure is performed in the trauma room at only 13/19 (68%) supraregional, 15/27 (56%) regional and 18/38 (47%) local trauma centers. The remainder of the supraregional and regional centers and 15/38 (39%) of the local centers stated that they have the required material and personnel who can perform a thoracotomy independently within and outside standard working hours. Table 4 shows the time until the person responsible for performing the intervention is available. It is clear that the time of arrival is not a limitation for the implementation (during regular working hours: supraregional, 0 min; regional, 5 min; local, 10 min; outside regular working hours: supraregional, 15 min; regional, 20 min; local 30 min; numerical value equals the median). The question of where the material is stored in these hospitals explains the discrepancy in the implementation of procedures in the trauma room, despite the provision of the material and human resources. All but one of the supraregional, one of the regional and two of the local trauma centers stated that they have the material available in the operating theatre.

### Indication for REBOA

A review by Morrison et al. reported that limb hemorrhage can be controlled by manual compression, pressure dressing, or tourniquets but that the vast majority of life-threatening hemorrhages occur in noncompressible body regions, such as the pelvis or abdomen. The mortality rate amonge patients who suffer such injuries is thus high (> 40%) [23, 24]. Balloon occlusion of the aorta is performed by 15/17 (88%) supraregional, 18/28 (64%) regional and 4/39 (10%) local trauma centers for a given emergency indication.

The efficacy of REBOA as a life-saving method in the context of shock in abdominal and pelvic hemorrhage has been described in several papers [25, 26].

### Treatment with external fixation

The discriminatory power of the question on the provision of external fixation systems for treating injuries to the extremities was not sufficiently selected, as some external fixators for fingers were listed, which are not the focus of emergency care in a MASCAL.

### Material supply

In a MASCAL, it may be useful for the rescue team to replenish relevant consumables in the emergency departments so that these consumables are readily available. Recommendations on this topic for the emergency department have been published [27]. The resulting additional demand for material must be taken into account when other actions are taken; otherwise, a bottleneck for additional material may occur in clinics. On the basis of the results of our survey, however, we cannot conclude to what extent individual clinics have considered this important factor in their preparations.

### Limitations

The information provided by the non-trauma-certified hospitals is not representative due to the low response rate compared to the response rate of the trauma centers.

## Conclusion

Although expert opinions and guidelines recommend and call for measures such as emergency thoracotomy, trepanation or aortic occlusion using the REBOA technique, these measures are not comprehensively implemented, as this study of Bavarian hospitals shows.

The results of this study call existing concepts into question and demonstrate a need for further research. An additional concrete question could be whether the care of patients by means of the abovementioned procedures in a MASCAL should be reserved for higher-level trauma centers or whether lower-level hospitals should be involved in the care.

## Data Availability

The datasets used and/or analysed during the current study are available from the corresponding author on reasonable request.

## Abbreviations

MASCAL: Mass casualty incident
TerrorMASCAL: Terror attack mass casualty incident
e.g.: For example
DGU: German Society of Trauma Surgery (Deutsche Gesellschaft für Unfallchirurgie)
US: United States
REBOA: Retrograde endovascular aortic occlusion
TC: Trauma center
NS: Neurosurgery
TS: Trauma surgery
ICP: Intracranial pressure
EVD: External ventricular drainage
STC: Supraregional trauma center
RTC: Regional trauma center:
LTC: Local trauma center
ASSET: Advanced surgical skills for exposure in trauma
PERT: Prehospitaland emergency department thoracotomy
